# Morphological and anatomical characterization of yellow diploid potato flower for effective breeding program

**DOI:** 10.1038/s41598-022-20439-6

**Published:** 2022-09-30

**Authors:** María de los Angeles Bohórquez-Quintero, Daicy Yaneth Galvis-Tarazona, Diana Marcela Arias-Moreno, Zaida Zarely Ojeda-Peréz, Sergio Ochatt, Luis Ernesto Rodríguez-Molano

**Affiliations:** 1 Grupo de Investigación BIOPLASMA-UPTC, Escuela de Ciencias Biológicas, Facultad de Ciencias, Universidad Pedagógica y Tecnológica de Colombia, Tunja, Colombia; 2 Agroécologie, INRAE, Insitut Agro, Université Bourgogne, Université Bourgogne Franche-Comté, 21000 Dijon, France; 3 Facultad de Ciencias Agrarias, Departamento de Agronomía, Universidad Nacional de Colombia, Carrera 30 Núm. 45-03, Edificio 500, Bogotá D.C., Colombia

**Keywords:** Developmental biology, Plant sciences

## Abstract

The diploid yellow potato (*Solanum tuberosum* L. Phureja Group) is an important plant genetic resource. In this study, we report for the first time the characterization of anther development and pollen formation in the cultivar Criolla Colombia. The description of morphological and histological characters of buds and flowers at different developmental stages permitted to identify ten main stages, from the differentiation of the male cells of the sporangium, meiosis, microspores formation and maturation, to the release of mature pollen. In addition, the results provide a graphic guide of the development of the anther, through the sequential and orderly formation of the epidermis, the endothecium, the middle layer and the nutritive layer or tapetum. This microanatomical information will be useful for work focused on androgenesis and identification of gene regulation in floral biology and gamete formation. Therefore, this study determined that to efficiently obtain haploids, flower buds between 5 and 8.9 mm long (stage 6 to 8) should be used, in which tetrads and microspores are in the early uninucleate and binucleate stage.

## Introduction

The Solanaceae family has been the center of important research advances for decades. It comprises 102 genera and 2500 species, with great abundance, distribution and endemism in Latin America^1,2^. In the Solanaceae, the genus *Solanum* stands out, with approximately 2000 species registered including the potato, the main non-cereal product commercialized globally^3,4^. In *Solanum*, the Phureja group consists mainly of diploid potatoes. These show a wide phenotypic diversity and important agronomic and nutritional characteristics^4,5^. Therefore, it is important to take advantage of its wild and commercial varieties in genetic improvement programs. The Creole potato or diploid yellow potato (*Solanum tuberosum* L. Phureja Group)^6^ corresponds to morphotypes that present tubers with yellow skin color and flesh^7^, and it constitutes one of the most important plant genetic resources in Colombia, the largest producer, consumer and exporter of diploid potato in the world^8^, due to its nutritional and organoleptic value^9,10^.

In Solanaceae, anther development and pollen grain formation have been described for *Cestrum bigibbosum* Francey^11^, species of the genus *Capsicum* L.^12^, *Petunia hybrida* Juss.^13^, *Solanum pimpinellifolium*^14^ and potato, among others. In *S. tuberosum*, microsporogenesis is known in three tetraploid somatic hybrids and their di(ha)ploid crosses^15^. The meiotic behavior of pollen mother cells has also been reported for potato, in relation to the ploidy level of somatic hybrids (*S. tuberosum* × *S. chacoense)*^12^, as also to the development of the anther, microsporogenesis and gametophyte formation^16^. However, these works are shallow and do not offer a detailed and extended descriptive and iconographic characterization of the process. As far as we know, no similar studies are known for creole potato.

It is essential to study the different aspects of pollen biology to understand the mechanisms of plant reproduction for a successful implementation of breeding programs, as they allow to increase the efficiency in the crossings, assuring an abundant production of seeds^17^. Pollen formation is a function of the stamen that takes place within the anther and involves numerous histochemical changes^18,19^. The stages of pollen development proceed in a sequential and orderly manner, and exhibit genetic and enzymatic coordination^20–22^. The analysis of the relationship between flower size and the stage of pollen development (microsporogenesis) provides an important tool for the induction of androgenesis^23^. This makes it possible to identify the stage or state in which the regeneration of haploid plants can be induced with greater efficiency^24,25^.

Doubled haploid production is a valuable biotechnology that can accelerate the breeding of new plant varieties by several years through the one-step creation of 100% homozygous plants^26^. Haploids can be induced in vitro via cultivating the haploid gametes or in vivo through inter- and intraspecific hybridization^27^. In addition, it facilitates the estimation of the quantity and quality of the pollen, to optimize hybridization processes in genetic breeding programs^28^. Therefore, the histological study of microsporogenesis offers opportunities for the control of fertility in plants of commercial interest^29^. Additionally, knowing the tissue conformation of the anther and the general morphology of the pollen can have taxonomic and systematic utility^11,16^.

There are studies on microsporogenesis^16^ and pollen meiosis and mitosis in *S. tuberosum*^30^. However, the different species and cultivars may present developmental particularities, which justifies the need for further research input on this subject^31^. In diploid yellow potato the ontogeny and histological development of pollen and anther and their correlation with flower formation^20^ still remain unknown, despite their commercial and agronomic relevance. Therefore, and considering that, the microspore is at the center of a variety of topics in modern plant science and breeding^32^, the aim of the present study was to characterize for the first time the pollen and anther formation, and to evaluate its association with anatomical changes in flower development in diploid yellow potato (*S. tuberosum* L. Phureja Group), cultivar Criolla Colombia; as a precise, fast, easy and reliable guide to identify microspores or pollen at development particular stages.

## Results

### Anatomical characterization of flower

**Figure 1:**
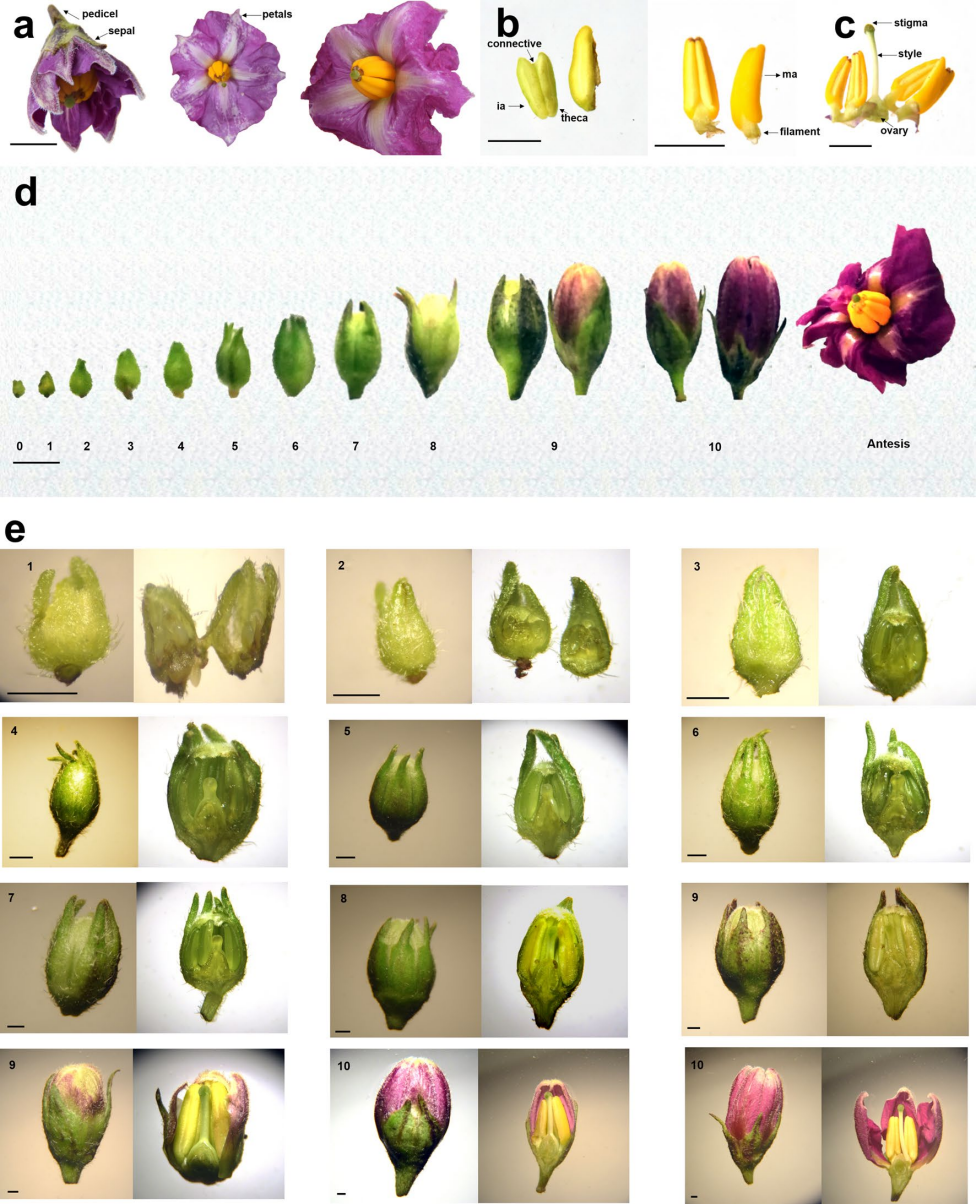
Buds and flowers of diploid yellow potato (*Solanum tuberosum* Grupo Phureja) cultivar criolla Colombia (scale bar **a–d** = 5 mm; **e** = 1 mm). Arabic numerals 1 to 10 represent anther developmental stages. Representative images of: (**a)** Perfect flowers with sepals, petals, anther ring and stigma. (**b**) Anthers. (**c**) Androecium and Gynoecium. (**d**) Development of buds from state 0 to 10. **(e)** Full bud and its cross section developing from state 0 to 10. *ia* immature anther, *ma* mature anther. Photographs and images created by the authors.

Criolla Colombia presents perfect, actinomorphic, pedicelate, whorled and pentamerous flowers. The androecium contains five exserted stamens inserted in the corolla; which, has a rotated shape, predominant coloration of intense lilac and secondary white, on both sides. The ovary is superior, the stigma is greenish in color and protrudes from the anther ring (Fig. 1a). The flowers are distributed in a terminal cymose inflorescence. The anther contains four lobes that are attached to the filament by connective tissue and together form the stamen (Fig. 1b,c). We found that flower development can be associated with ten main stages (Fig. 1d,e), for buds between 0.1 and 15 mm.

### Anther and pollen development

**Figure 2:**
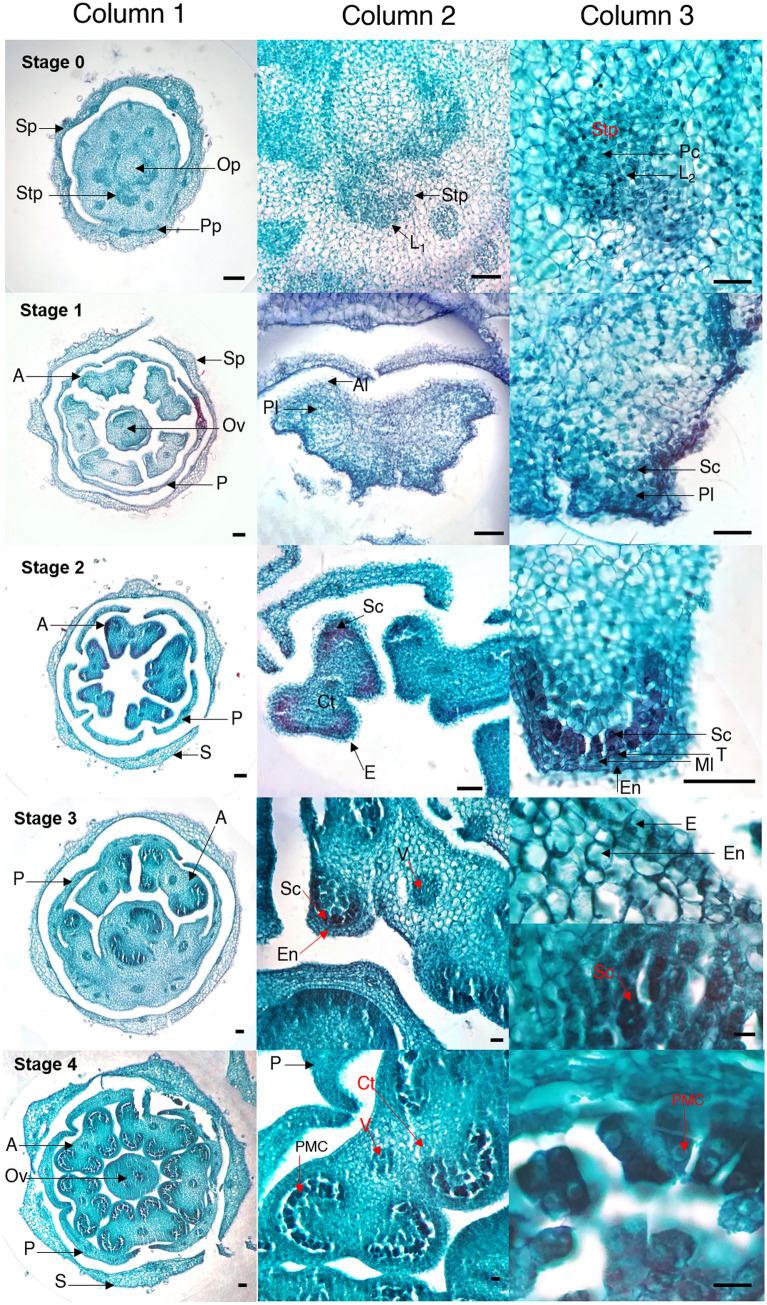
Anther development and pollen formation in diploid yellow potato (*Solanum tuberosum* Phureja Group) Criolla Colombia cultivar. Stages of development: 0–4. *A* Anther, *Al* anther lobe, *Ct* connective tissue, *E* epidermis, *En* endothecium, *L1, L2* lines of primordia of the stamen, *Ml* midline, *Ov* ovary, *Op* ovary primordia, *P* petal, *Pc* procambial cells, *Pl* parietal line, *PMC* pollen mother cell, *Pp* petal primordia, *Sc* sporangial cells, *Sp* sepal primordia, *S* sepal, *Stp* stamen primordia, *T* tapetum, *V* vascular tissue. Scale bars, 100 µm (column 1); 50 µm (column 2); 25 µm (column 3). Image created by the authors.

**Figure 3:**
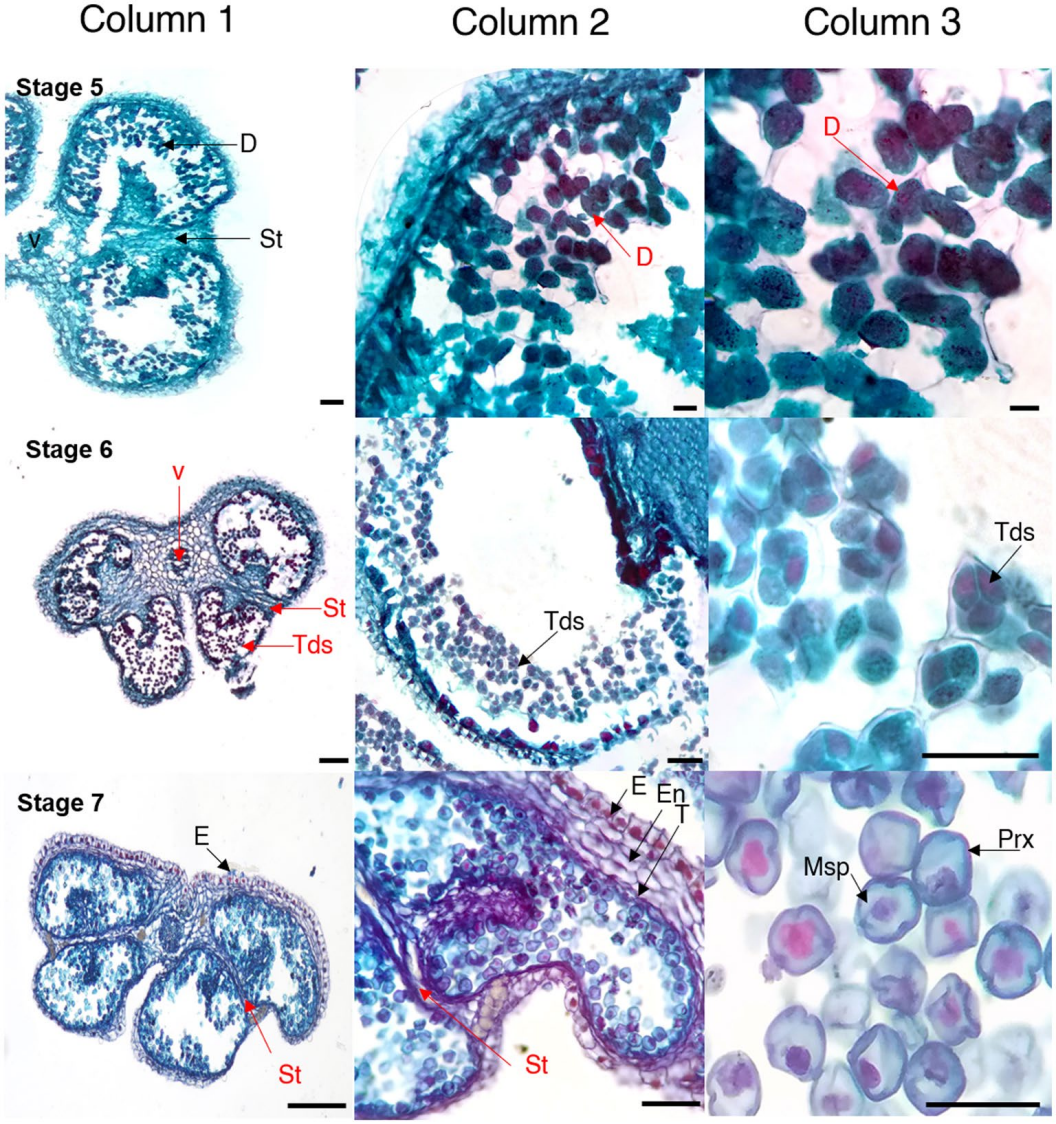
Anther development and pollen formation in diploid yellow potato (*Solanum tuberosum* Phureja Group) Criolla Colombia cultivar. States of development: 5–7. *Dy* Dyads, *E* epidermis, *En* endothecium, *Msp* microspore, *Prx* primexin, *St* septum, *T* tapetum, *Tds* tetrads, *V* vascular tissue. Scale bars, 100 µm (column 1); 50 µm (column 2); 25 µm (column 3). Image created by the authors.

**Figure 4:**
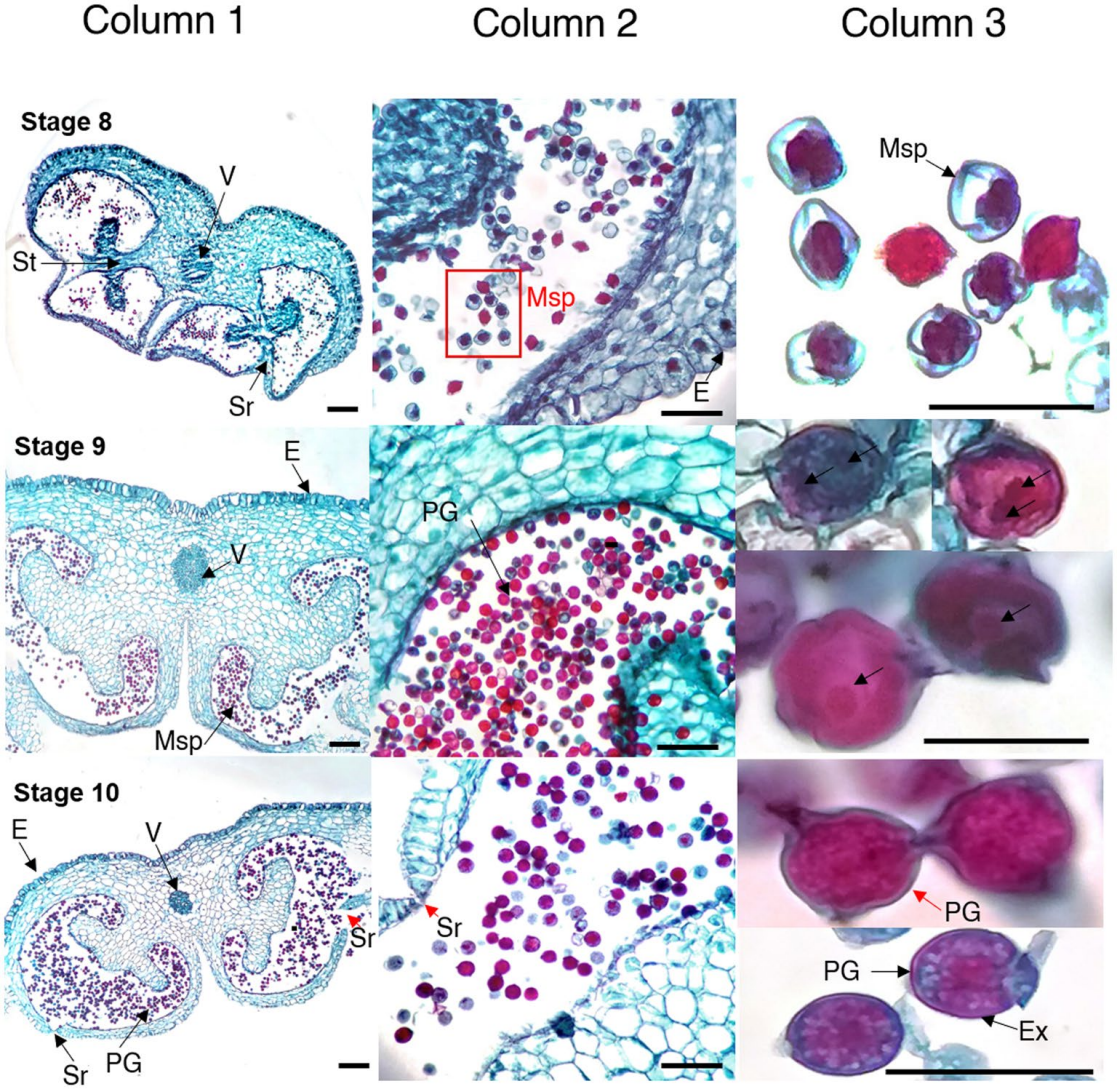
Anther development and pollen formation in diploid yellow potato (*Solanum tuberosum* Phureja Group) Criolla Colombia cultivar. States of development: 8–10. *E* epidermis, *Ex* exine, *Msp* microspore, *PG* pollen grain, *Sr* stomium region, *St* septum, *V* vascular tissue. Scale bars, 100 µm (column 1); 50 µm (column 2); 25 µm (column 3). Image created by the authors.

Pollen development within the anther depends on joint interactions between gametophytic and sporophytic tissues. This process includes a series of crucial phases such as differentiation of male cells from the sporangium, meiosis, formation and maturation of the microspore, and finally release of mature pollen (Figs. 2, 3, and 4).

We describe for the first time the correlation between bud/anther size and the progression of microspore development for cultivar Criolla Colombia. The landmark morphometric and histological macro- and micro-anatomical events for each developmental stage were determined (Table 1; Figs. 2, 3 and 4). The data in Table 1 underline the relevance of undertaking the microscopic observations added to the phenological ones to ascertain the precise microsporogenetic stage of flower buds, given the small differences observed in their size. Additionally, the statistical analysis determined that these differences between the development stages for length and diameter of buds and anthers are significant (Kruskal–Wallis p < 2.2e−16; Table S1-S6).

**Table 1:** Stages of anther and pollen development in diploid potato cultivar Criolla Colombia (*Solanum tuberosum* Phureja Group).

Stage	Bud	Anther		Landmark stage of development
	Length $\overline{X }$± SD $(\mathrm{mm})$	Length $\overline{X }$ ± SD $(\mathrm{mm})$	Diameter $\overline{X }$± SD $(\mathrm{mm})$	
0	0.912 ± 0.040	0.225 ± 0.038	0.086 ± 0.008	Primordia
1	1.425 ± 0.125	0.488 ± 0.105	0.260 ± 0.022	Formation of anther lobes and primary parietal and sporangia cells
2	1.842 ± 0.115	0.796 ± 0.158	0.454 ± 0.037	Layers: epidermis, endothecium, midline, and tapetum
3	2.666 ± 0.175	1.566 ± 0.271	0.652 ± 0.024	Sporangial cells
4	3.616 ± 0.215	2.411 ± 0.147	0.859 ± 0.023	Pollen mother cell
5	4.596 ± 0.303	2.755 ± 0.090	1.039 ± 0.022	Dyads
6	5.618 ± 0.205	3.566 ± 0.205	1.256 ± 0.026	Tetrads
7	7.093 ± 0.234	4.445 ± 0.258	1.456 ± 0.024	Young microspores
8	8.232 ± 0.366	5.458 ± 0.264	1.664 ± 0.022	Late microspores
9	10.173 ± 0.442	6.330 ± 0.121	1.844 ± 0.022	Mature pollen
10	13.308 ± 1.156	6.864 ± 0.095	2.052 ± 0.021	Anther dehiscence: pollen release

The results showed significant differences between all the development stages, for length and diameter of buds and anthers, according to Kruskal–Wallis and Dunn’s tests.

*SD* standard deviation.

### Stage 0

Buds shorter than 1 mm and light green in color (Fig. 1d,e). At the histological level, primordia of sepals, petals, stamens, filaments, and ovary were observed and, in turn, dividing cells within these (Fig. 2-Stage 0). Within the primordium of the stamen, the preliminary formation of two cell layers and procambial cells was distinguished. L1, cells that will form the epidermis; L2, which will give rise to germ cells, endothecium, midline and tapetum, and procambial cells derived from a previously formed L3 layer, which will develop vascular and connective tissue^33,34^.

### Stage 1

Flower buds between 1 and 1.5 mm long and partially light green in color. Sepals larger than the buds (Fig. 1d,e). The anthers began to acquire a tetrasporangial shape, due to the continuous cell division in each of the lobes (Fig. 2-Stage1).

### Stage 2

Bud length ranged from 1.6 to 2 mm. The bud was green and formation of petals was observed, below which the developing stamens are located (Fig. 1d,e). Sporangial cells covered the lobular space, and the epidermis, endothecium, medial layer, and tapetum were distinguished in the anther (Fig. 2a-Stage 2). In addition, sporophytic cells, located in the U-shaped region, were also identified.

### Stage 3

The buds were between 2.1 and 2.9 mm long and increased their volume. In addition, anthers covered by the greenish petals that were still united were observed, and early development of the gynoecium was observed (Fig. 1d,e). In the general structure of the bud, histologically, sepals, petals (located outside the stamens), anthers and ovary were distinguished. The cells of the sporangium were distinguishable (Fig. 2-Stage 3).

### Stage 4

Buds with a length between 3 and 3.9 mm. In this stage, an increase in the diameter of the bud and in the size of the anther, ovary, style, and stigma were observed. The union of the petals in the apical part was observed to be densely covered by trichomes (Fig. 1d,e). Histologically, the Pollen Mother Cells (PMC) began to separate from each other^25^ (Fig. 2-Stage 4).

### Stage 5

The length of the bud was between 4 and 4.9 mm. The base of sepals was colored with anthocyanins. Larger petals, anthers and style were observed. The gynoecium acquired a whitish hue on the outside (Fig. 1d,e). The PMC began their first meiotic division, forming two dyads (chromosomal load n). Likewise, at this stage the septum and also amorphous tapetum cells were observed (Fig. 3-Stage 5).

### Stage 6

The buds were between 5 and 5.9 mm long, with thinner sepals towards the terminal part. The external-terminal coloration of the petals was whitish, and the length of the anthers increased (Fig. 1d,e). The stigma became round (Fig. 3-Stage 6). The occurrence of Meiosis II gave rise to the development of four haploid cells, known as tetrads or tetraspores.

### Stage 7

The length of the buds was between 6 and 7.4 mm. An increase in the size of the anthers was observed, which covered the entire space of the bud. In the anther, the connective of the theca and the apical part of the sepals were demarcated, it was observed in purple color (Fig. 1d,e). From the tetrads, haploid microspores were released. In these, the formation of a large vacuole was observed and, in the periphery, primexine was observed, which serves as a scaffold for pollen wall formation^35^ (Fig. 3-Stage 7).

### Stage 8

The buds measured between 7.5 and 8.9 mm. The size of the petals (whitish) became slightly larger than that of the sepals (dark green). The style and stigma showed white and green coloration, respectively. In the anthers (green-yellowish) an increase in size was noticeable (Fig. 1d,e). Histologically, the beginning of the resorption of the septum and the beginning of the integration of the two locules within each theca was observed. In microspores, the nucleus was displaced by the vacuole to one end and wall thickening occurred (Fig. 4-Stage 8).

### Stage 9

The length of the buds ranged from 9 to 10.9 mm. The petals showed lilac and white coloration in the basal and apical parts, respectively. The pistil and ovary increased in size. The color of anthers changed from green to yellow (Fig. 1d,e). Histologically, in the bilobed anther, the external locules (near the petals) were larger than the internal ones (near the pistil). In each of the haploid microspores formed during meiosis, the nucleus divided by asymmetric mitosis and developed a bicellular pollen grain, distinguishing the vegetative and germinative pores^12^ (immature male gametophyte) (Fig. 4-Stage 9).

### Stage 10

Buds between 11 and 15 mm long, petals larger than the sepals and predominantly violet in color (with anthocyanin pigments). This stage occurs prior to anthesis, so the petals begin to separate at the apical part of the flower. The yellow anthers formed a ring on which the pistil was distinguished (Fig. 1d,e). In addition, anther dehiscence due to stomium cell degeneration was observed. Finally, three germinal openings were identified in the pollen grains and consequently their release occurred (Fig. 4-Stage 10).

**Figure 5:**
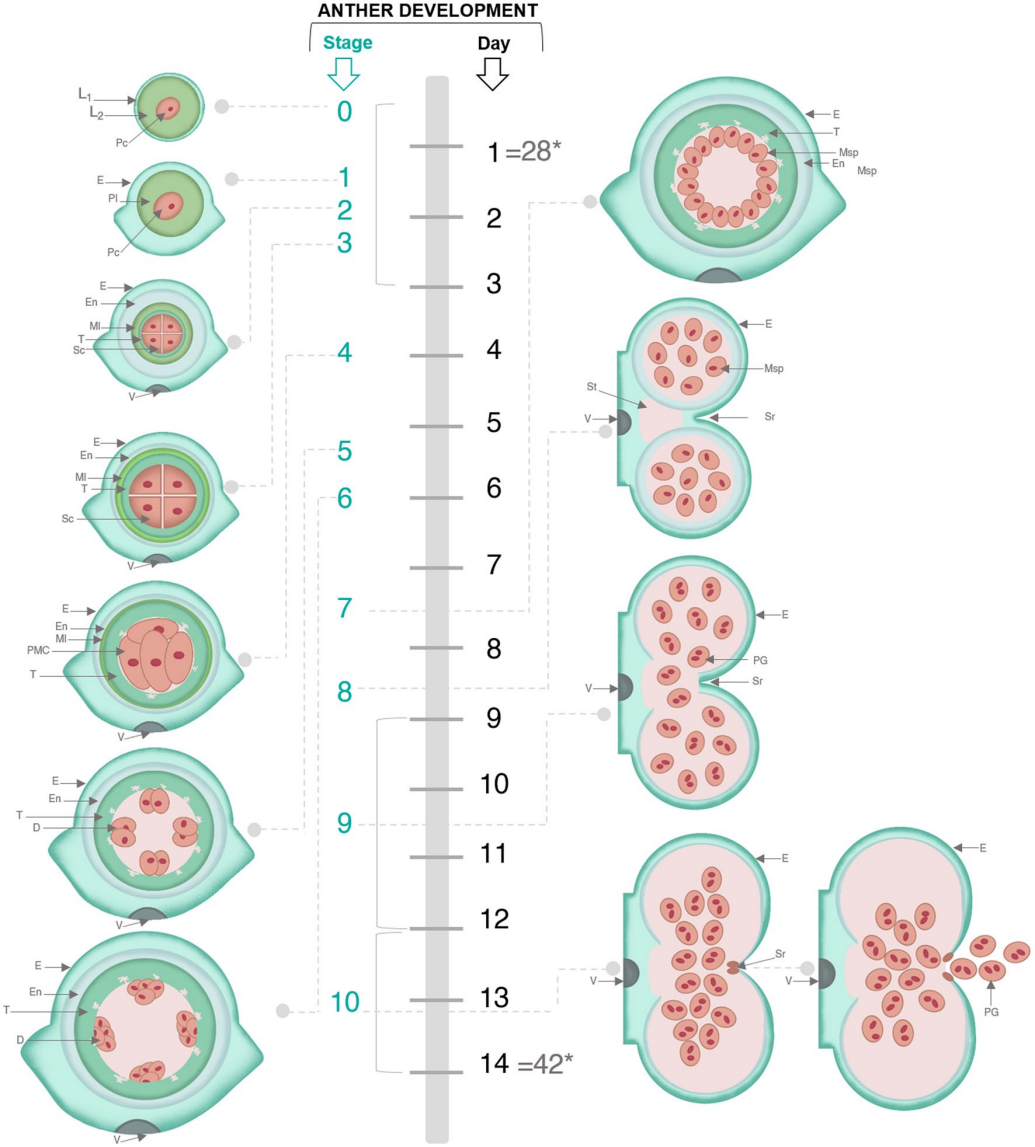
Schematic view of formation of diploid yellow potato anther cell layers. The diagram shows the relationship between the stages of flower bud development and the duration expressed in days. The left side of the figure details the first six stages (0 to 6) of anther development; while the right side of the image shows stages 7 to 10. *D* Dyads, *E* epidermis, *En* endothecium, *L1, L2* lines of primordia of the stamen, *Ml* midline, *Msp* microspore, *Pc* procambial cells, *PG* pollen grain, *Pl* parietal line, *PMC* pollen mother cell, *Sc* sporangial cells, *Sr* stomium region, *St* septum, *T* tapetum, *Tds* tetrads, *V* vascular tissue. Asterisk: corresponds to the number of days after emergence. Scheme devised and recreated by the authors with images illustrated by Camila Reyes and Angie Carvajal.

In addition, it was observed that in the diploid yellow potato crop, flowering is staggered and can last up to 80–85 days after planting. There are usually two flowering cycles; the first occurs on main stems, while the second is characteristic of secondary stems. Flower buds < 5 mm are identified approximately 28 days after emergence (crop emergence occurs approximately 20 days after tuber planting and corresponds to the appearance of the first leaves on the soil surface). While buds between 12 to 14 mm (stage 10) are observed 42 days after emergence (Fig. 5). An open flower can last 2–4 days depend on environmental conditions.

**Figure 6:**
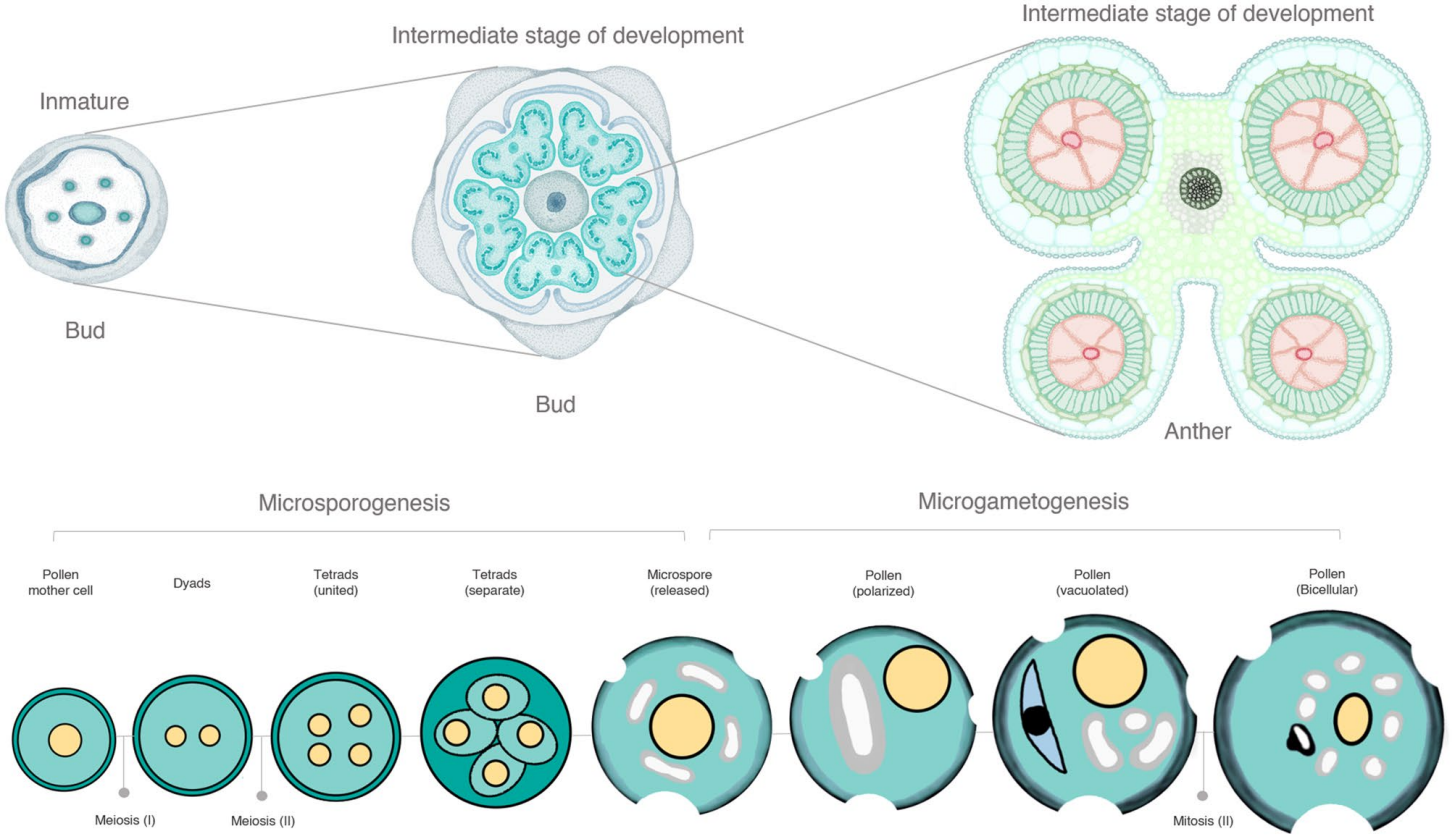
Schematic view of cytological events associated with development of bud, anther y pollen in diploid yellow potato. Scheme devised and recreated by the authors with images illustrated by Camila Reyes and Angie Carvajal.

Finally, based on these observations, it could be suggested that the anther and pollen development period of diploid yellow potato involves 10 to 14 days (Fig. 5). This process is illustrated by means of a scheme that includes the main histological and cytological events associated with the anther and pollen development process (Fig. 5 and 6). Considering the relation between the stages of development and the duration expressed in days.

## Discussion

The characterization of anther and pollen development, as well as the mechanisms of pollen release, are essential to understand the formation of embryos and seeds in most angiosperm species^29,36^. In this study, it was shown that the morphological and descriptive characterization of the flower for cultivar Criolla Colombia agree with those described for different *Solanum* species, suggesting a pattern of similar correspondence within the genus^37^*.*

Pollen is the male gametophyte that carries plant genetic information and its study is relevant since the adequate male germline development is a prerequisite for proper crops performance^38,39^. Pollen development is a critical step in plant development that is needed for successful breeding and seed formation^29^. In this study, the microsporogenesis from the differentiation of the archeosporial cells to the maturation of the pollen was described for the cultivar Criolla Colombia through ten stages. This process has also been referenced for other Solanaceae such as cherry tomato (*Solanum pimpinellifolium*)^14^, tomato (*Solanum lycopersicum*)^40^ and tobacco (*Nicotiana tobaccum*)^41^, in terms of the formation of anthers and male gametophytes.

Like other angiosperms, in the development of Criolla successful anther/microspore culture largely depends on the use of microspores at appropriate developmental stages at the time of culture, which can be specific for each plant species and genotype^42^. Here, the correlation between morphological characteristics, such as floral bud length and diameter and anther length, and specific microspore developmental stages is described for the first time, for cultivar Criolla Colombia. The findings of the present work suggest that flower buds with lengths between 5 and 8.9 mm (stage 6), in which tetrads and microspores are in the uninucleate and early binucleate stages, should be used for the induction of androgenesis, as also observed in several other species^31,40,43,44^. In this study, these cytological phases were observed within the characterization of the formation of male gametes, for the first time in *Solanum tuberosum* L. Phureja. So, towards the middle of the process, the first meiotic division occurred and with it the formation of dyads; followed by meiosis II, which gave rise to the development of tetrads (stage 6 to 8). Subsequently, the presence of vacuolated haploid microspores (stage 7) that underwent mitosis, giving rise to immature gametophytes (stage 8), was distinctive. In addition, in the last stages, the typical morphology of the pollen was identified, with its corresponding germinative pores.

In Criolla Colombia, pollen formation began with the differentiation of archeosporial cells into primary parietal and sporogenous cells^45^; which gave rise to the four typical lobes of the anther morphology. Within each lobe, the epidermis, the endothecium, the middle layer and the tapetum were identified. These layers play important roles in microsporogenesis. Thus, for example, the epidermis provides structural support, protects and prevents water loss in the anthers. The endothecium, together with the epidermis, increases the thickness of the outer wall of the lobe and provide mechanical force for the dehiscence of the anther^16^. The middle layer completely surrounds the pollen sac and, together with the tapetum, performs secretory and nutritional functions during pollen development^16^.

The histological micropreparations obtained here also allowed to identify the septum, a layer that separates the locules within each theca in the anther, and whose reabsorption gave rise to the integration of a single locule per theca. Likewise, in this study the intine and exine pollen layers were notable in haploid microspores and mature pollen, respectively. On the other hand, it was distinctive that with the reabsorption of the stomium, the anther acquired the anatomical characteristics necessary for its opening and the subsequent release of mature pollen^46^.

Additionally, it is important to note that the observations allowed to identify microspores in different stages of development within and between anthers. So that, the processes involved in microsporogenesis may not be synchronous within a flower, an anther, and even a microsporangium^42^. Asynchrony appears to be normal and has been documented for several species^31,42,47^. This could cause metabolic differences between developing microspores and thus physiologically heterogeneous pollen grains^48^. These events may have an adaptive role, since more efficient pollen grains would be selected in terms of homeostasis during pollen tube growth, tolerance to desiccation, resilience, speed of (re)hydration and germination^48^.

Likewise, the histological events of anther and microspore formation characterized in this study, agree with those described for different *Solanum* species^14–16,49^. This research consolidated a precise and non-invasive method to correlate morphological markers with microspore/pollen grain formation stages for cultivar Criolla Colombia. This will guide the selection and collection of flower buds/anthers harboring microspores at particular stages of development^31,42,50^. Therefore, this new knowledge should be used within in vitro culture for the isolation, storage and use of anthers or microspores, for the evaluation of pollen viability or vigor and/or for obtaining double haploids^19^. It is well known that, within breeding programs, obtaining double haploids is one of the greatest limitations^51^, and these findings would allow its accelerated improvement to efficiently obtain diploid, self-compatible and 100% homozygous plants^52^.

This research describes jointly and for the first time the development of the buds, the anther, and the pollen in potatoes. As such, the information presented in this study is of great relevance not only for creole potato crop but also for other commercially important Solanaceae. The results obtained are expected to strengthen the fundamental and applied agronomic research as well as commercial growth of this important phytogenetic resource, of high internal demand and great export potential^53^.

## Conclusion

This work offers the first macro- and micro-anatomical report of anther and pollen development in *Solanum tuberosum* L. Phureja, in general, and for Criolla Colombia potato in particular. Our results extend the knowledge on the identity and differentiation of microsporogenesis in this species of great economic importance. The histo-cytological events of the ten stages of pollen and anther development detailed here could be the basis for the formulation of identification studies on gene regulation in floral biology and gamete formation, as well as to obtain haploids and double haploids of utility in breeding programs.

## Methods

### Plant material

*Solanum tuberosum* L. Phureja Group, Cultivar Criolla Colombia, is a native cultivar type, the result of a clonal selection process within the population of genotypes with yellow round tuber^54^.

The research was carried out at the BIOPLASMA Plant Tissue Culture Laboratory of the Faculty of Science within the Universidad Pedagógica y Tecnológica de Colombia. All procedures were conducted in accordance to the guidelines**.** For this study, samples of plant material were supplied by Luis Ernesto Rodríguez-Molano researcher who obtained the registration of the cultivar criolla Colombia as member of the potato research group of Faculty of Agronomy, within the Universidad Nacional de Colombia.

Inflorescences with buds and flowers in different stages of development were stored immediately maintaining the cold chain (5 °C) from the separation of the plant to their processing in the laboratory. Buds, flowers, and anthers representative of each developmental stage were photographed for illustrative purposes. In addition, to know the time required by flower buds to convert from one stage to another, we follow the flowering process in the crop.

### Anatomical and metric characterization of the flower

The floral development in diploid yellow potato cultivar Criolla Colombia was described from the appearance of flower buds to full bloom (more than 75% of open flowers), using qualitative descriptors proposed by Navarro et al*.* (2010)^55^ and Gómez (2000)^56^ as a guide. These included degree of flowering, shape of the corolla, color of the flower, predominant color, intensity of predominant color, secondary color, distribution of secondary color, pigmentation in anthers, pigmentation in the pistil, color of the calyx and color of the pedicel.

The length and diameter of buds (n = 250) and anthers (n = 250) were measured. The results for each stage of development were expressed as the mean ± standard deviation. Given that not all data showed a normal distribution, the non-parametric Kruskal–Wallis (P < 0.05) test followed by Dunn’s test was used, instead of analysis of variance (ANOVA), to compare the stages for each variable measured.

### Histological characterization

For each developmental stage, anthers were isolated and fixed for 24 h in FAA (50% ethanol, 5.0% glacial acetic acid, 3.7% formaldehyde). Subsequently, the samples were dehydrated in a series of ethanol (50, 60, 70, 80, 96, 3 × 100 v/v, 3 h per step), isopropanol and acetone (100 v/v, 1 h). Flower buds and anthers were embedded in paraffin, and histological sections (8–12 µm) were obtained with a rotary microtome. Cross-sections were stained with Safranin-FastGreen and observed under a microscope. These were selected and used in the microstructure analysis. The stages of microspore/pollen development were characterized according to reference studies^14,16,57,58^^.^. For each stage twenty micropreparations (technical replicates) were observed, the best being selected to graphically represent the development of microsporogenesis.

### Statement stating procedures according on plant ethics

All procedures were conducted in compliance with specific norms and regulations. For this study, samples of plant material were supplied by Luis Ernesto Rodriguez-Molano researcher (co-author of this paper) who obtained the registration (PAP-05-39) of the cultivar criolla Colombia as member of the potato research group of Faculty of Agronomy, within the Universidad Nacional de Colombia. The registration and complementary information related to this cultivar can be consulted through the link https://www.papaunc.com/catalogo/criolla-colombia or in book^59^ and article^54^.

## Supplementary Information

Supplementary Tables.

## Data Availability

The datasets generated and/or analyzed during the current study are available online in “Link Supporting data—Bohórquez et al. 2022”.
