# Supplementary material for: Morphological and anatomical characterization of yellow diploid potato flower for effective breeding program
Source: Sci Rep. 2022 Sep 30;12:16402. doi: 10.1038/s41598-022-20439-6 (PMC9525687; doi:10.1038/s41598-022-20439-6)

**Morphological and anatomical characterization of yellow diploid potato flower for effective breeding program**

Scientific Reports

María de los Angeles Bohórquez-Quintero^1^ ([Orcid:](https://orcid.org/0000-0002-2887-4389) [0000-0003-1919-4346](https://orcid.org/0000-0003-1919-4346)), Daicy Yaneth Galvis-Tarazona^1^ ([Orcid:](https://orcid.org/0000-0002-2887-4389) [0000-0002-2251-0015](https://orcid.org/0000-0001-6171-0549)), Diana Marcela Arias-Moreno^1*^([Orcid:](https://orcid.org/0000-0002-2887-4389) [0000-0001-6171-0549](https://orcid.org/0000-0001-6171-0549)), Zaida Zarely Ojeda-Peréz^1^ ([Orcid:](https://orcid.org/0000-0002-2887-4389) [0000-0003-1630-6699](https://orcid.org/0000-0003-1630-6699)), Sergio Ochatt^2^ ([Orcid: 0000-0002-2887-4389](https://orcid.org/0000-0002-2887-4389)), Luis Ernesto Rodríguez Molano^3^ (Orcid: 0000-0002-9058-8404)

^1^Grupo de Investigación BIOPLASMA-UPTC, Facultad de Ciencias Básicas, Universidad Pedagógica y Tecnológica de Colombia. Avenida Central del Norte 39-115, Tunja, Boyacá, Colombia.

^2^Agroécologie, INRAE, Institut Agro, Univ. Bourgogne, Univ. Bourgogne Franche-Comté, F-21000 Dijon, France.

^3^Facultad de Ciencias Agrarias, Departamento de Agronomía, Universidad Nacional de Colombia, Carrera 30 núm. 45-03, Edificio 500, Bogotá D. C., Colombia.

* Corresponding author. e-mail: [diana.arias04@uptc.edu.co](mailto:diana.arias04@uptc.edu.co).

**BUTTON LENGTH**

**Table S1** The non-parametric Kruskal–Wallis test (p< 0.05) for *Button Length.*

**
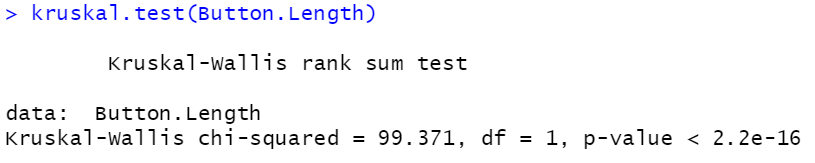
**

**Table S2** The Dunn’s test used to establish how many stages were significantly different from each other, for *Button Length.*

**
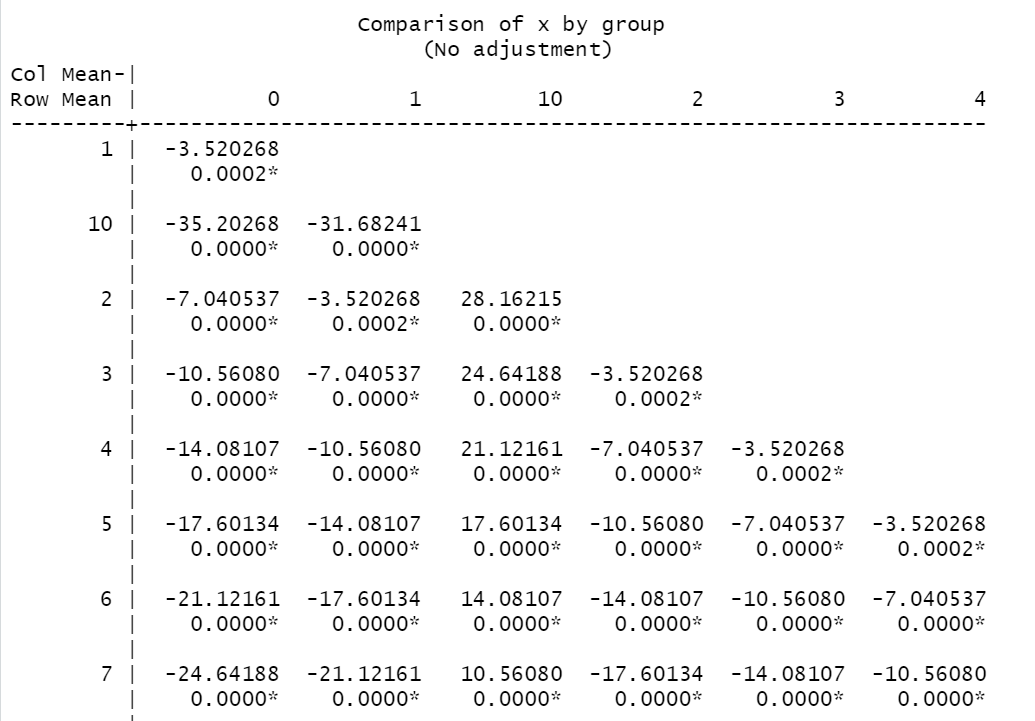
**

**
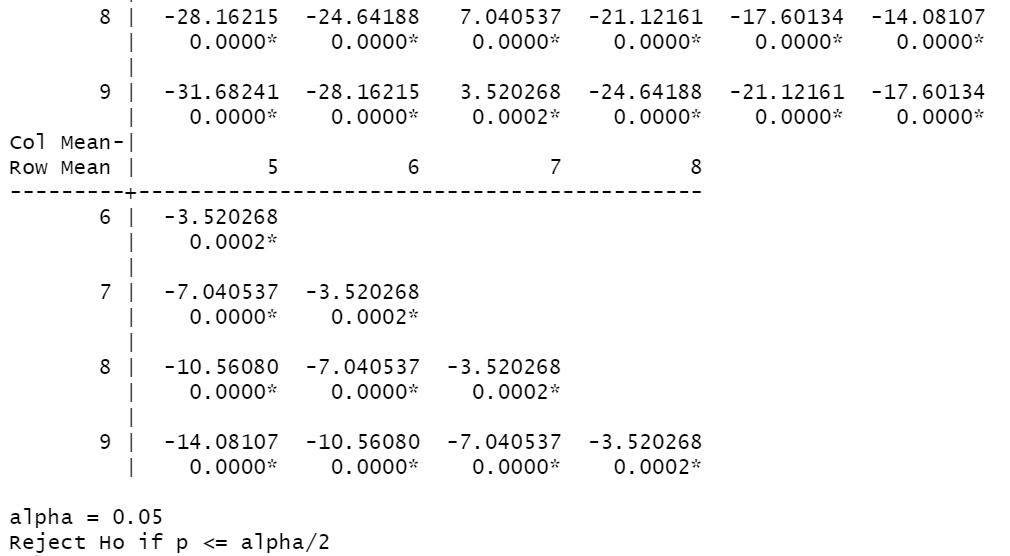
**

**ANTHER DIAMETER**

**Table S2** The non-parametric Kruskal–Wallis test (p< 0.05) for *Anther Diameter.*

**
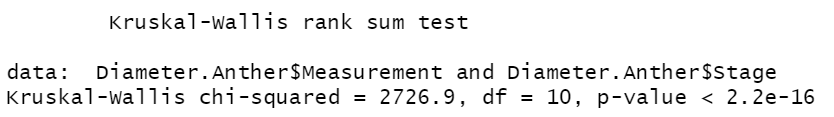
**

**Table S3** The Dunn’s test used to establish how many stages were significantly different from each other, for *Anther Diameter.*


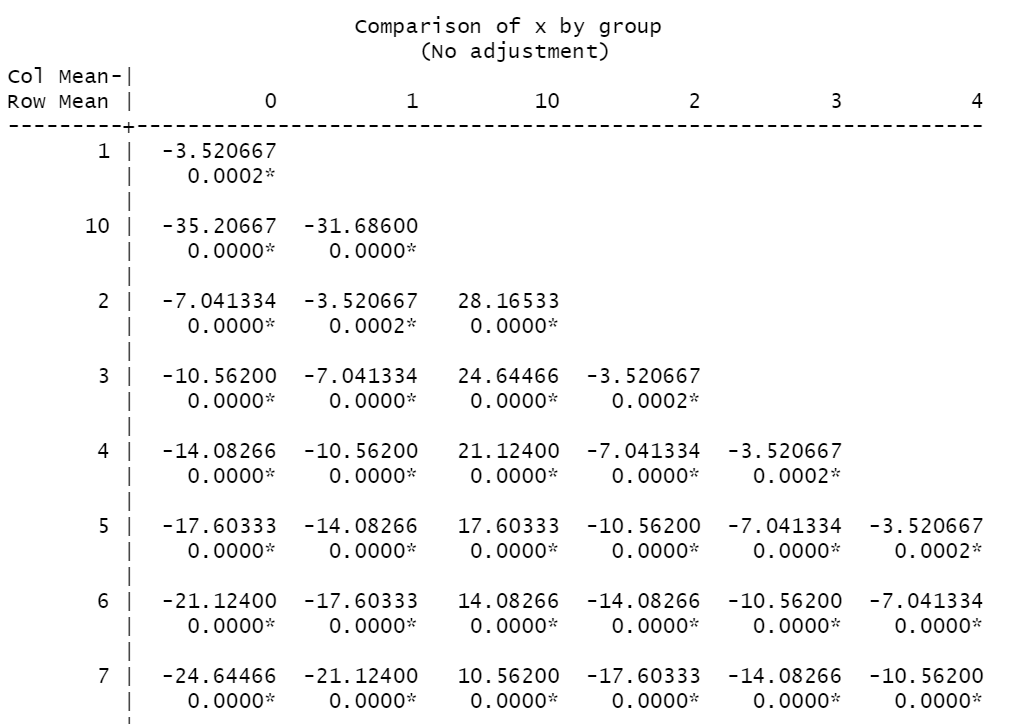


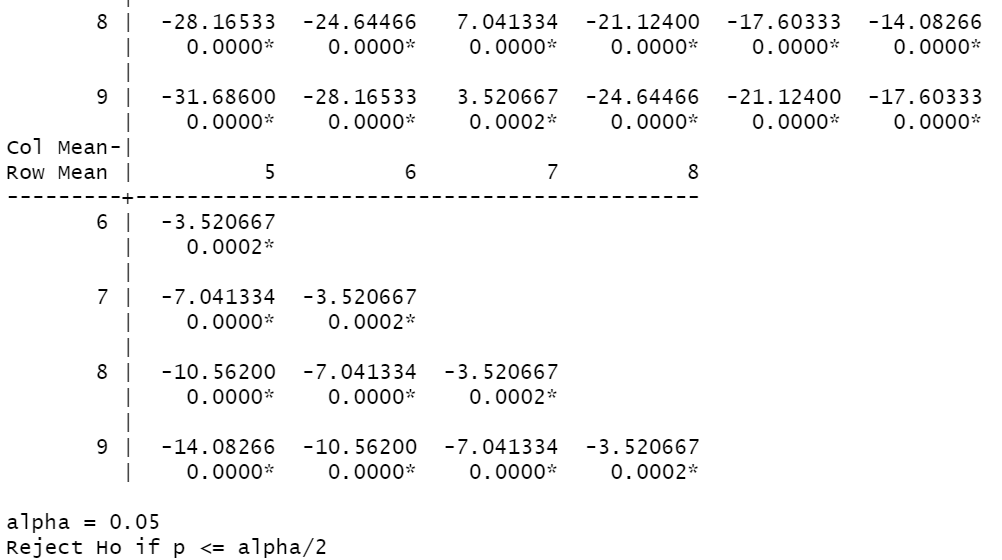


**ANTHER LENGTH**

**Table S4** The non-parametric Kruskal–Wallis test (p< 0.05) for *Anther Length.*

**
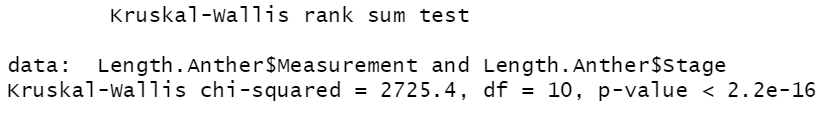
**

**Table S5** The Dunn’s test used to establish how many stages were significantly different from each other, for *Anther Length.*


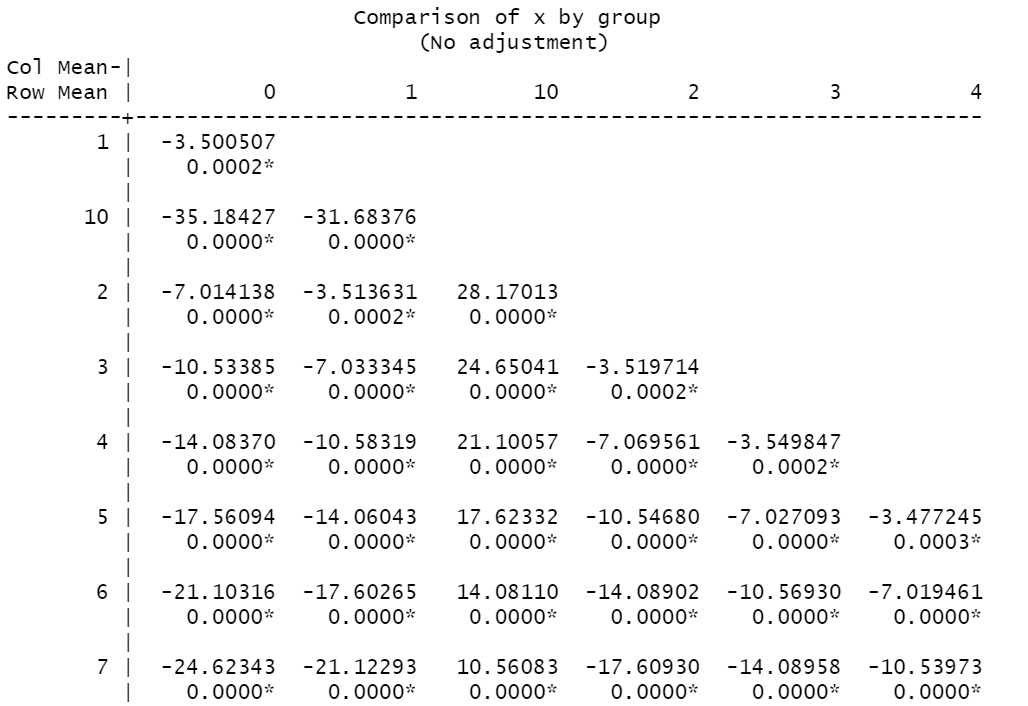


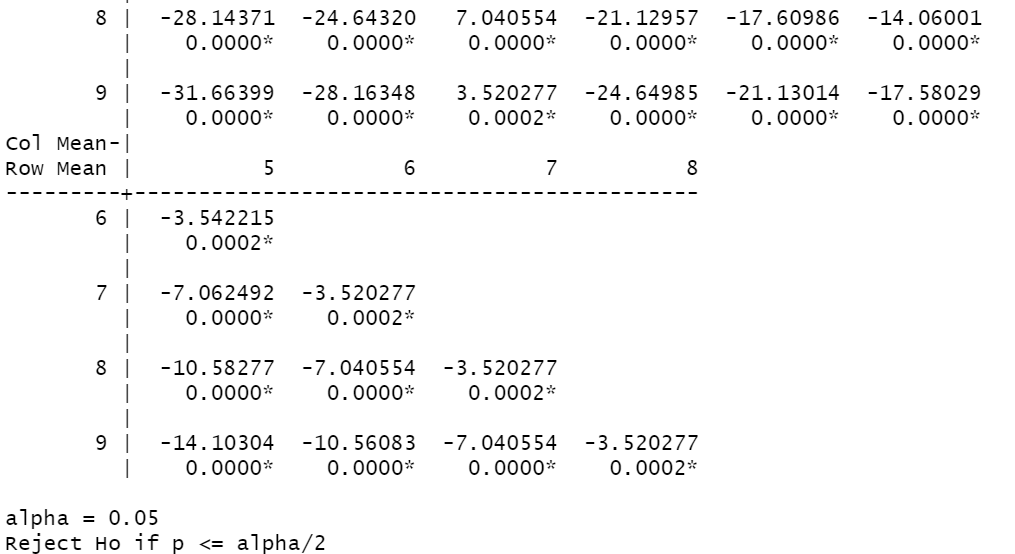

Supplement: Supplementary file 1 — Supplementary Tables. [file 41598_2022_20439_MOESM1_ESM.docx]
